# Predictors of Preterm Birth among Mothers Who Gave Birth in Silte Zone Public Hospitals, Southern Ethiopia

**DOI:** 10.1155/2021/1706713

**Published:** 2021-02-24

**Authors:** Jida Ali Hassen, Mengistu Nunemo Handiso, Bitiya Wossen Admassu

**Affiliations:** ^1^Public Health Department, Silte Zone Health Department Disease Prevention and Control Coordinator, Silte Zone, Ethiopia; ^2^Public Health Department, College of Medicine and Health Sciences, Wachemo University, Addis Ababa, Ethiopia; ^3^Jimma University School of Public Health, Reproductive Health and Nutrition Department, Jimma, Ethiopia

## Abstract

**Background:**

A preterm birth is the leading cause of death in both neonatal and children under five years of age every year throughout the world, particularly in Sub-Saharan Africa. The causes of a preterm birth are complex and multifactorial; many risk factors that contribute it are not fully understood. The aim of this study was to identify predictors of a preterm birth among mothers who gave birth in Silte Zone Public Hospitals, Southern Ethiopia (2019/20).

**Methods and Materials:**

A hospital-based unmatched case-control study design was carried out from July 15^th^ to October 30^th^, 2019, by assigning mothers who gave preterm births as cases and those with term births as controls. A total of 365 respondents (91 cases and 274 controls) were selected by a consecutive simple random sampling until the required sample size was achieved. For each case, three consecutive controls were included. Data were collected using a structured interview questionnaire complement with record reviewing. The data were entered into Epi Info 7 and exported into SPSS 25 for analysis. Descriptive analysis was computed to obtain summary values for cases and controls separately. All candidate variables in bivariate analysis were entered into the multivariable logistic regression model by using the backward likelihood ratio selection methods. Finally, variables with *p* value ≤ 0.05 were considered as potential determinants of a preterm birth and reported in the form of adjusted odds ratio with 95% confidence interval.

**Results:**

Among a total of 365 mothers who gave live birth, 91 (24.9%) were cases compared to 274 (75.1%) which were controls. The final multivariable logistic regression analysis results showed that having history of a previous preterm birth (AOR = 3.51; 95%CI = 1.40 − 8.81), having shorter interpregnancy interval (AOR = 4.46; 95%CI = 1.95 − 10.21), experiencing obstetric complication (AOR = 3.82; 95%CI = 1.62 − 9.00), and having infant born with low birth weight (AOR = 5.58; 95%CI = 2.39 − 13.03) were found to be independent predictors of a preterm birth.

**Conclusions:**

According to this finding, mothers having previous history of a preterm birth, experiencing obstetric complication, having shorter interpregnancy interval, and having infant born with low birth weight were reported as the independent predictors of a preterm birth. Improving the quality of antepartum and intrapartum, counseling on birth space, creating awareness on family planning, and early screening of preterm determinants are mandatory.

## 1. Introduction

A preterm birth is a syndrome with a variety of causes and underlying factors, usually divided into spontaneous and provider-initiated preterm births. A doctor recommends premature childbirth at the initiative of a health professional and suggests early childbirth if the baby or mothers have a health problem. A spontaneous preterm birth is a birth that begins on its own and that happens when a woman enters premature labor or when her water breaks too early. Most preterm births occur spontaneously, but some are caused by the early induction of labor or caesarean section, whether for medical or nonmedical reasons [1, 2].

Ethiopia is also one of the major countries with the highest number of under-five deaths and newborns worldwide in 2016. It represents 3.3% and 3.4% respectively of all under-five and newborn deaths in the world. From 320,000 babies born prematurely in Ethiopia each year, an estimated 24,400 (7.6%) deaths of children under five years of age are caused by the direct effects of a premature complication [3–5]. Again the causes of prenatal mortality in Ethiopia are compiled from hospital-based data, among obstetric complications; also the third highest identified cause was prematurity of 7%, and interventions can be directed at all women for primary prevention and reduction of the risk of a preterm birth or used to minimize the risk in pregnant women with known risk factors [6, 7].

In developing countries, the attention given to a preterm birth is less. In Ethiopia especially, little is known about determinants that contribute to a preterm birth and also previous studies were focused mainly on the magnitudes, rather than its determinants. Despite the burden of a preterm birth, few effective strategies or approaches are available to prevent a preterm birth and reduce related deaths. In some parts of Ethiopia, limited studies show that premature childbirth is critical, while those in Southern Ethiopia, particularly in the current study area, are not being studied.

Therefore, the aim of this study was to identify determinants of a preterm birth at Silte Zone Public Hospitals, Southern Ethiopia. This will help health providers to have adequate knowledge and intervene early in the antenatal care visit and also might have an input to programmers and policy makers.

## 2. Methods

### 2.1. Study Setting

A facility-based unmatched case-control study was conducted at Silte Zone Public Hospitals, Southern Ethiopia from July 15^th^ to October 30^th^, 2019. Cases were defined as all selected mothers who gave a live preterm birth (<37 0/7 weeks), whereas controls were also defined as all selected mothers who gave live from 37 weeks and above of gestation (≥37 0/7weeks). However, gestational age was used to determine both cases and controls based on either by last menstrual period (LMP) or early ultrasound (before 24 weeks of gestation) assessments. All selected mothers who gave live (<37 0/7 weeks) for case and term birth (≥37 0/7weeks) for control were considered as the study population.

Mothers who have multiple births, postpartum psychosis, seriously ill, unable to communicate, and referred immediately to other health institution did not know their LMP and have no early ultrasound assessment, and mothers referred to the facility without antenatal information were excluded.

### 2.2. Sampling Methods and Procedure

The sample size was determined by two population proportion formulas and it calculated using Stat Calc Epi Info 7.1.4 software considering the following statistical assumptions: 95% confidence level (*Z*_*α*/2_ = 1.96), 80% power of the study (*Z*_*β*_ = 0.84), and ratio of control to case 3 : 1 (*r* = 3). Different exposure variables that have high contribution for a preterm birth were selected from related previous studies and the main exposure variable that gives highest sample size was taken. Based on this, place of rural residence was chosen as an independent variable since it gave maximum sample size as compared to other exposure variables. 31.8% of mothers gave live full-term births (assuming the control group was exposed), when rural residence was a risk factor for a preterm birth (AOR = 2.13) [8]. After adding 10% nonresponse rate, the required sample size was 365 mothers (91 cases and 274 controls).

Study participants were selected by consecutive simple random sampling techniques until the sample sizes were accomplished from all four public hospitals in Silte Zone, after proportional allocation for each hospital based on considering average of a three-month expected skilled delivery (2,495) report in 2018/19 year prior to the data collection period. Once a mother with a preterm birth was recruited as a case group, the next three consecutive mothers were selected as the control groups at the same condition in respective hospital and recruitment was continued until the required sample size is fulfilled. In case a selected mother did not fit the criteria or declined to consent, the next mother was selected.

### 2.3. Data Collection Methods and Procedure

Eight data collectors and three supervisors qualified in diploma and BSc midwife, respectively, were assigned for each of the selected hospitals after two consecutive days of training was given. Data were collected through a face-to-face interview by using pretested structure questionnaires, and also review maternal and neonatal medical charts were checked to take and confirm for some important variables within 48 hours after they gave birth. The interview, document review, and physical measurements were conducted by trained midwife nurses working in each hospital. Data were collected from participants after they were fully recovered from delivery process, who are ambulant, stable, and fit for interview and measurements almost within 48 hours after giving birth. The overall estimated time to take information from each mother was needed for almost 25-30 minutes.

### 2.4. Measurements

Physical measurements were obtained by using anthropometric measurement equipment following standardized techniques for each mother and newborn infant after birth. Maternal weight was measured using weight scale with height rod (Italy). The instrument was calibrated each time before use with a standard one-kilogram weight material. A mother puts still in the middle of scale stand without touching anything and with the body weight equally distributed on both feet. The weight was taken to the nearest 100 g (0.1 kg). Maternal height was also measured using scale with height rod (Italy), while the mother was in standing position, barefooted with heels together, arms hanging naturally at the sides, legs straight, and shoulders relaxed. The maternal mid-upper arm circumference (MUAC) was measured using a flexible nonstretchable standard tape to the nearest 0.1 cm. The circumference was measured at the midpoint between the tip of the acromion process of the scapula and olecranon process of the ulna. Similarly, newborn weight was also measured using a balanced digital SECA scale (Germany) which is a nonhanging type. The scale was always calibrated using a standard one-kilogram weight material, and the reading on each scale was taken to zero level before weighing each newborn. The measurements were taken to the nearest 100 grams. All measurements were taken twice with the same measuring instrument, and the average was computed. Gestational age in weeks was estimated either by LNMP or early ultrasound assessment through interview and taking from documentation to the nearest of 0/7 week birth expressed date of completed weeks.

### 2.5. Data Management and Analyses

The questionnaires were checked in the field manually for completeness and consistencies before data entry. Data were entered into Epi Info 7.1.4 software and exported to Statistical Package for Social Sciences (SPSS) version 25 software for analyses after editing, coding, and cleaning. Descriptive statistics were employed to describe the characteristics of the study participants in relation to outcome variable by using crosstabulation expressed as frequency and percentage for the case and control groups, respectively.

Both bivariate and multivariate analyses were employed by using logistic regression model to identify determinants of a preterm birth that are statistically significant. Since the interest is in identifying determinants of a preterm birth, the dependent variables are coded as 1 if the mother is with a preterm birth and coded as 0 if not.

First, simple binary logistic regression analysis was run to identify crude association between dependent variable and each of independent variables that were statistically significant at *p* value ≤ 0.25 with 95% CI and crude odds ratio (COR) were considered for the final multivariable analysis. Subsequently, those identified candidate variables in bivariable analysis were incorporated into the multivariable logistic regression model by using the backward stepwise elimination likelihood ratio (LR) selection methods in order to identify the independent predictors of a preterm birth among the study participants.

Appropriate model diagnostics tests were assessed whether the required assumptions for the application of multivariable logistic regression analysis were fulfilled. Multicollinearity test was performed to see the presence of collinearity among independent variables by using a standard error prior to determining the final model. Results with inflated standard error > 2 were excluded from the analysis. And also the goodness-of-fit test was checked using the Hosmer-Lemeshow test statistics (*p* value = 0.407) to check the appropriateness of model for analysis. The final findings of the model were reported by using AOR with 95% CI at *p* value ≤ 0.05 as a cut-off point to be considered statistically significant. Furthermore, results were summarized and displayed using tables, figures, and graphs.

### 2.6. Data Quality Control

A two-day training was given to data collectors and supervisors prior to the start of the survey objective data collection process. The area of training was how to interview study participants about the use of measurement scales, allowable criteria and ethical issues, and how to correctly complete the questionnaire. During the training, participants read the questionnaire and subsequent discussions were held on the entire content of the questionnaire and the problem areas were adjusted.

Similarly, the questionnaire was pretested on 5% of total sample size of the study (18 mothers) in Worabe Health Center, which is related to the client visit for maternal care services. Based on the findings and experiences from the pretest modification on logical sequence, simplicity and clarity of the question were done.

Furthermore, data collectors checked the questionnaires for completeness before leaving each study participant. Both supervisors and investigators made close follow-up and reviewing each questionnaire regularly to check for completeness, consistency, and clarity in each hospital. Finally, before data analysis editing, coding, cleaning, and data exploration were done.

### 2.7. Ethical Consideration

Ethical clearance was obtained from Wachemo University College of Medicine and Health Science ethical review committee to carry out this study. A formal support letter was written from Wachemo University to Silte Zone Health Departments. Also, a formal letter of permission was secured before starting data collection process from Silte Zone Health Departments to each of the selected public hospitals. An informed verbal consent was obtained from the mothers after fully explaining the purpose of the study throughout the data collection period. Participants were informed about the right not to participate in or withdraw from the study at any time through the interviews. All the information obtained were kept confidential at all time by using codes and were collected anonymously.

## 3. Results

### 3.1. Sociodemographic Characteristics of Mothers

Among a total of 365 mothers who gave single live birth in Silte Zone Public Hospitals, 91 (24.9%) were the case groups compared to 274 (75.1%) which were the control groups; this makes a response rate of 100%.

Regarding the sociodemographic characteristics, the majority of mothers (64 (70.3%) of the case groups and 222 (81%) of the control groups) were found in the age range of 20-34 years. About 55 (60.4%) of the case group and 157 (57.3%) of the control group mothers were living in rural area. Again concerning the marital status of mothers, about 74 (81.3%) of the case groups and 248 (90.5%) of the control groups were married. With regard to religious and ethnic status, majority of 78 of the case groups (85.7%) and 251 (91.6%) of the control groups were Muslim and 74 (81.3%) of the case groups and 242 (88.2%) of the control groups were Silte, respectively (Table 1).

### 3.2. Obstetric and Gynecologic Characteristics of the Study Participant

Multigravida and multiparous mothers were comprised nearly similar proportion in both the case groups (70 (76.9%) and 68 (74.7%)) and the control groups (210 (76.6%) and 204 (74.5%)), respectively. Concerning history of immediate previous pregnancy outcome, 33 (47.1%) of the case groups and 27 (12.9%) of the control groups had previous history of a preterm birth. Similarly, a proportion of previous history of still birth, abortion, and congenital anomalies in the case groups were 6 (8.6%), 11 (15.7%), and 6 (8.6%), while in the control groups were 14 (6.7%), 25 (11.9%), and 10 (4.8%), respectively (Table 2).

### 3.3. Maternal Preexisting Medical Disorders

Regarding human immunodeficiency virus (HIV) status, majority (80 (87.9%)) of the case groups and (250 (91.2%)) of the control groups were tested for HIV infection; however, 6 (7.6%) of the case groups and 14 (5.6%) of the control groups were positive for HIV infection. With regard to anemia status, approximately 90% of mothers in both the case and control groups were screened for hemoglobin level, from which majority of mothers (66 (81.5%)) of the case groups and (222 (88.1%)) of the control groups had their hemoglobin level 11 gm/dl and above (Table 3).

### 3.4. Neonatal Characteristics

**Figure 1:**
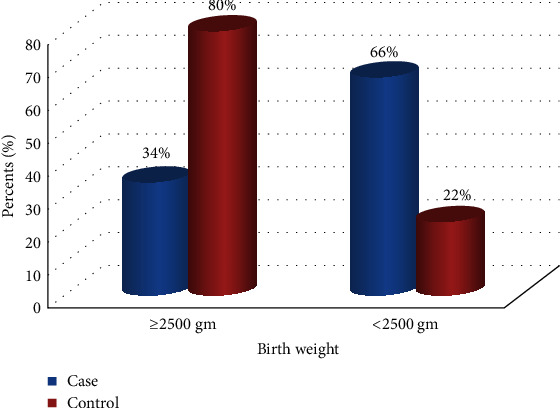
Distribution of neonatal birth weight had born among mothers who gave birth in Silte Zone Public Hospitals, Southern Ethiopia (2019/20).

Nearly above of the average (50 (54.9%)) of the case groups and (144 (52.6%)) of the control groups were female sex. Regarding the presentation during delivery, infants delivered in vertex presentation were higher among in the control groups (254 (92.7%)) than those in the case groups (81 (89%)). Similarly, about below the half (41 (45.1%)) of the case group and (52 (19%)) of the control group neonates experienced birth asphyxia after birth. Furthermore, the proportion of low birth weight (<2500 gm) was higher among the case group (60 (65.9%)) than that of the control group (55 (20.1%)) (Figure 1).

### 3.5. Determinants of Preterm Births

Bivariate analysis showed that monthly family income, history of a preterm birth, interpregnancy interval, ANC visit, mode of delivery in current pregnancy, experiencing obstetric complication in current pregnancy, maternal weight, infant birth weight, and presence of birth asphyxia were candidate variables for multivariable analysis at *p* value ≤ 0.25 and have crude association with the occurrence of a preterm birth and also collectively entered to multivariable logistic regression model using the backward elimination stepwise likelihood ratio selection method to identify determinants.

After adjusting for covariate, the multivariable logistic regression analysis showed that having history of a previous preterm birth, having shorter interpregnancy interval (<24 months), having any one or more obstetric complications (such as PIH, APH, PROM, and poly/oligohydramnios), and having infants with low birth weight (<2500 gm) were found to be independent predictors for the occurrence of a preterm birth at *p* value ≤ 0.05.

Accordingly, the odds of developing a preterm birth were 3.5 times more likely among mothers who had history of a previous preterm birth as compared to mothers who had no previous history of preterm births (AOR = 3.51; 95%CI = 1.40 − 8.81; at *p* value = 0.007). In the same manner, those mothers who had shorter interpregnancy interval (<24 months) were about 4.5 times more likely to develop a preterm birth compared to mothers with recommended interpregnancy interval (≥24 months) (AOR = 4.46; 95%CI = 1.95 − 10.21; at *p* value ≤ 0.001).

Additionally, mothers who experienced any one or more obstetric complications (such as PIH, APH, PROM, and poly/oligohydramnios) during the current pregnancy had 4 times higher odds of developing preterm births as compared to those who were not experienced any one or more obstetric complications at all (AOR = 3.82; 95%CI = 1.62 − 9.00; at *p* value = 0.002). Furthermore, the odds of developing a preterm birth were 5.6 times more likely among newborn who had low birth weight (<2500 gm) as compared to those newborn who had normal birth weight (≥2500 gm) (AOR = 5.58; 95%CI = 2.39 − 13.03; at *p* value ≤ 0.001) (Table 4).

## 4. Discussion

This study identified both maternal- and neonatal-related factors had a significant association with the occurrence of a preterm birth among mothers who gave birth in the study area.

Accordingly, mothers who had previous history of a preterm birth were more likely to deliver a preterm birth compared to mothers who had no previous history of a preterm birth. This finding is in agreement with previous studies conducted in Northern, Southwest, and Southeast Ethiopia and found that previous history of a preterm birth increases the risk of a preterm birth [8–10] and also this fact is relevant to other studies done in Nairobi, Kenya; in Rabat, Morocco; and in Southern Nigeria, which showed that the chance of delivering a preterm birth was significantly higher among mothers who had previous history of a preterm birth [10–12]. Similarly, this result is consistent with previous studies conducted in Ardabil, Iran; in Lima, Peru; and in Thanavur, India, that the previous history of a preterm birth potentiates the risk of having a consequent preterm birth [13, 14]. The exact mechanism for this determinant has not been well understood and is also unable to explain but this might be due to the combined effects of other persistent determinants precipitating a preterm birth subsequently.

On the other hand, this study found that mothers with shorter interpregnancy interval (<24 months) had higher odds of bearing a preterm birth than mothers with the recommended interpregnancy interval (≥24 months). This finding is in line with report from different studies done in Axum and Adwa, Northern Ethiopia; Jimma, Southwest Ethiopia; and Amhara Regional State of Ethiopia, in which mothers with shorter interpregnancy interval increase the risk of a preterm birth [8, 10, 15, 16]. This result is also supported with other studies conducted in Rabat, Morocco, and in Tehran, Iran, in which mothers who had a shorter interpregnancy interval were among predictors that increase the chance of a preterm birth [11, 13]. This could be due to the impacts on mothers' health and having insufficient time to establish a proper anatomical, physiological, and psychological balance and recovery after past pregnancy which adversely affects fetal growth as a result of causing a narrow birth space.

Additionally, this study revealed that mothers experienced any one or more obstetric complications (included PIH with its complication such as preeclampsia/eclampsia, APH due to placenta previa/abruption, and PROM and poly/oligohydramnios) in current pregnancy had high risk of having a preterm birth than those mothers without these obstetric complications. This finding correlates to the finding of studies conducted in Debre Markos, Debre Tabor, and Hosanna Town, Northern and Sothern Ethiopia, respectively, which showed that mothers having obstetric complication increase the risk of having a preterm birth [17–19]. This report was also in conjunction with previous related studies carried out in different settings such as in Enugu State, Nigeria, and in Dar es Salaam, Tanzania, which conclude that mothers experiencing obstetric complication during pregnancy have increased risk of having a preterm birth [20, 21]. This might be attributed to the occurrence of pregnancy-induced hypertension and antepartum hemorrhages that can be predisposed to an earlier medically initiated preterm birth as an intervention strategy for any one of these cases, while premature rapture of the membranes or poly/oligohydramnios by itself activates preterm labor.

The odds of developing a preterm birth were more likely to occur among newborn who had low birth weight (<2500 gm) as compared to their counterparts. This result is in agreement with a study carried out in Northern Ethiopia, which revealed that infants with LBW were more likely to have a preterm birth [16]. Similarly, this result is also supported with other studies carried out in Northeastern Tanzania; in Porto Alerge, Brazil; and in Western Iran and found that LBW had significantly associated with a preterm birth [22, 23]. This is possible to the interchangeable relationship between fetal weights and gestational ages and indirectly shortens the intrauterine development time, and because of that, problem identified and incorporated earlier may also prevent oxygen and nutrients essential to reach the fetus across the placenta. After that, further future studies might need to realize the mechanism of the observed association.

## 5. Strengths and Limitations of This Study

The most important strength of this study includes the following: it is conducted for the first time and done in all public hospitals in the study area to identify determinants of a preterm birth by using a case-control study. However, it is also carried out by using the combination of face-to-face interview and medical chart review for complementation and confirmation of some important variables, which is used to minimize the possible recall bias. This study also has some limitations: it might be a possibility of prone to recall bias because mothers were asked about their previous obstetric historic characteristics such as recall for gestational age based on their last date of menstruation, interpregnancy interval months, number of ANC visit, and months of iron-folate tablet taken, In order to minimize potential recall bias and to complete the interview medical record review for some important variables was used.

Again, in this study, it was not possible to enroll mothers who had difficulty to estimate their gestational age with unknown last normal menstrual period (LNMP) and missed the ultrasound result in their chart, which means to estimate gestational age not including other options like clinical assessment. Furthermore, this study is unable to include mothers who delivered at home because there might be a possibility of introducing selection bias.

## 6. Conclusions

This study showed that the independent predictors of a preterm birth were some maternal and neonatal bases to the occurrence of a preterm birth. Mothers having previous history of a preterm birth, experiencing any one or more obstetric complications, having shorter interpregnancy interval, and having infants delivered with low birth weight were reported as the most important maternal and neonatal predictors of a preterm birth. The essential strategies should be stressed on improving antenatal care services and promoting early detection and clinical managements which are crucial for those pregnant mothers experiencing any obstetric complications during their expected antepartum and intrapartum care service providing times. In addition to this, encouraging mothers to utilize contraceptive and providing health education on a preterm birth and obstetric complications may lead to decline the burden of a preterm birth and its consequences.

## Figures and Tables

**Table 1:** Sociodemographic characteristics of mothers who gave birth in Silte Zone Public Hospitals, Southern Ethiopia (2019/20).

Variables	Category	Case (*N* and %)	Control (*N* and %)
Age in years	≤19	07 (7.7)	16 (5.8)
	20-34	64 (70.3)	222 (81)
	≥35	20 (22)	36 (13.1)
Residence	Rural	55 (60.4)	157 (57.3)
	Urban	36 (39.6)	117 (42.7)
Marital status	Married	74 (81.3)	248 (90.5)
	Single	09 (9.9)	15 (5.5)
	Divorced/widowed	08 (8.8)	11 (4)
Religion	Muslim	78 (85.7)	251 (91.6)
	Orthodox	11 (12.1)	16 (5.8)
	Protestant	02 (2.2)	07 (2.6)
Ethnicity	Silte	74 (81.3)	242 (88.3)
	Gurage	09 (9.9)	18 (6.6)
	Hadiya	03 (3.3)	09 (3.3)
	Amhara	05 (5.5)	05 (1.8)
Educational status	No formal education	41 (45.1)	125 (45.6)
	Attended 1-8 grade	22 (24.2)	80 (29.2)
	Attended 9-12 grade	16 (17.6)	46 (16.8)
	College and above	12 (13.2)	23 (8.4)
Occupation	Housewife	40 (44)	138 (50.4)
	Government employee	15 (16.5)	35 (12.8)
	Self-employed	14 (15.4)	36 (13.1)
	Merchant	13 (14.3)	44 (16.1)
	Farmer	09 (9.9)	21 (7.7)
Estimated monthly family income (in ETB)	<1651	18 (19.8)	50 (18.2)
	1651-3200	34 (37.4)	62 (22.6)
	3201-5250	24 (26.4)	82 (29.9)
	5251-7800	12 (13.2)	44 (16.1)
	≥7801	03 (3.3)	36 (13.1)
Family members (in number)	<4 members	29 (31.9)	79 (28.8)
	≥4 members	62 (68.1)	195 (71.2)

**Table 2:** Obstetric and gynecologic characteristics of mothers who gave birth in Silte Zone Public Hospitals, Southern Ethiopia (2019/20).

Variables	Category	Case (*N* and %)	Control (*N* and %)
Gravidity	Primigravida	21 (23.1)	64 (23.4)
	Multigravida	70 (76.9)	210 (76.6)
Parity	Primipara	23 (25.3)	70 (25.5)
	Multipara	68 (74.7)	204 (74.5)
History of preterm birth	Yes	33 (47.1)	27 (12.9)
	No	37 (52.9)	183 (87.1)
History of stillbirth	Yes	06 (8.6)	14 (6.7)
	No	64 (91.4)	196 (93.3)
History of abortion	Yes	11 (15.7)	25 (11.9)
	No	59 (84.3)	185 (88.1)
History of congenital anomaly	Yes	6 (8.6)	10 (4.8)
	No	64 (91.4)	200 (95.2)
Interpregnancy interval (in months)	<24	34 (48.6)	30 (14.3)
	≥24	36 (51.4)	180 (85.7)
Previous history of mode of delivery	SVD	52 (74.3)	160 (76.2)
	Assisted	8 (11.4)	27 (12.9)
	CS	10 (14.3)	23 (11)
ANC follow-up in current pregnancy	Yes	81 (89)	263 (96)
	No	10 (11)	11 (4)
ANC visit (in number of visit)	<4	45 (55.6)	90 (34.2)
	≥4	36 (44.4)	173 (65.8)
Iron-folate supplementation in current pregnancy	Yes	70 (76.9)	221 (80.7)
	No	21 (23.1)	53 (19.3)
Iron-folate supplementation (in months)	<3	27 (38.6)	77 (34.8)
	≥3	43 (61.4)	144 (65.2)
Onset of labor in current pregnancy	Spontaneous	64 (70.3)	209 (76.3)
	Induced	27 (29.7)	65 (23.7)
Mode of delivery in current pregnancy	SVD	57 (62.6)	204 (74.5)
	Assisted	09 (9.9)	32 (11.7)
	CS	25 (27.5)	38 (13.9)
History of obstetric complication in current pregnancy	Yes	54 (59.3)	66 (24.1)
	No	37 (40.7)	208 (75.9)
PIH	Yes	14 (25.9)	15 (22.7)
	No	40 (74.1)	51 (77.3)
APH	Yes	15 (27.8)	18 (27.3%)
	No	39 (72.2)	48 (72.7)
PROM	Yes	24 (44.4)	29 (43.9)
	No	30 (55.6)	37 (56.1)
Poly/oligohydramnios	Yes	11 (20.4)	18 (27.3)
	No	43 (79.6)	48 (72.7)

**Table 3:** Preexisting medical disorder and physical and lifestyle characteristics of mothers who gave birth in Silte Zone Public Hospitals, Southern Ethiopia (2019/20).

Variables	Category	Case (*N* and %)	Control (*N* and %)
HIV testing	Yes	80 (87.9)	250 (91.2)
	No	11 (12.1)	24 (8.8)
HIV status	Negative	73 (82.4)	236 (94.4)
	Positive	06 (7.6)	14 (5.6)
Hemoglobin testing for anemia	Yes	81 (89)	252 (92)
	No	10 (11)	22 (8)
Hemoglobin level (g/dl)	<11.0	15 (18.5)	30 (11.9)
	≥11.0	66 (81.5)	222 (88.1)
History of UTI	Yes	14 (15.4)	33 (12)
	No	77 (84.6)	241 (88)
History of malaria attack	Yes	09 (9.9)	19 (6.9)
	No	82 (90.1)	255 (93.1)
History of chronic medical illness	Yes	16 (17.6)	30 (10.9)
	No	75 (82.4)	244 (89.1)
Presence of hypertension	Yes	11 (68.8)	21 (70)
	No	5 (31.3)	09 (30)
Presence of diabetes mellitus	Yes	06 (37.5)	10 (33.3)
	No	10 (62.5)	20 (66.7)
Maternal weight (kg)	<50	17 (18.7)	27 (9.9)
	≥50	74 (81.3)	247 (90.1)
Maternal height (cm)	<150	12 (13.2)	20 (7.3)
	≥150	79 (86.8)	254 (92.7)
Maternal MUAC (cm)	<23	60 (65.9)	166 (60.6)
	≥23	31 (34.1)	108 (39.4)
History of alcohol intake	Yes	04 (4.4)	06 (2.2)
	No	87 (95.6)	268 (97.8)
History of chat chewing	Yes	10 (11)	37 (13.5)
	No	81 (89)	237 (86.5)
History of taking medication	Yes	10 (11)	25 (9.1)
	No	81 (89)	249 (90.9)
History of work loaded	Yes	49 (53.8)	140 (51.1)
	No	42 (46.2)	134 (8.9)

**Table 4:** Bivariate and multivariable logistic regression analysis for determinants of PTB among mothers who gave birth in Silte Zone Public Hospitals, Southern Ethiopia (2019/20).

Variables and category	Case (*N* and %)	Control (*N* and %)	COR (with 95% CI)	AOR (with 95% CI)
Monthly family income (in ETB)				
<1651	18 (19.8)	50 (18.2)	1	1
1651-3200	34 (37.4)	62 (22.6)	*1.52* (*0.77-3.01*)	2.62 (0.85-8.08)
3201-5250	24 (26.4)	82 (29.9)	0.81 (0.40-1.65)	0.61 (0.19-2.02)
5251-7800	12 (13.2)	44 (16.1)	0.76 (0.33-1.75)	0.85 (0.22-3.36)
≥7801	03 (3.3)	36 (13.1)	*0.23* (*0.06-0.84*)^@^	0.14 (0.02-1.03)
History of preterm birth				
Yes	33 (47.1)	27 (12.9)	*6.04* (*3.25-11.23*)^@^	*3.51* (*1.40-8.81*)^∗∗^
No	37 (52.9)	183 (87.1)	1	1
Interpregnancy interval (months)				
<24	34 (48.6)	180 (85.7)	*5.67* (*3.08-10.40*)^@^	*4.46* (*1.95-10.21*)^∗∗∗^
≥24	36 (51.4)	30 (14.3)	1	1
ANC visit in current pregnancy				
<4	45 (55.6)	90 (34.2)	*2.40* (*1.45-3.99*)^@^	1.19 (0.51-2.74)
≥4	36 (44.4)	173 (65.8)	1	1
Mode of delivery in current pregnancy				
SVD	57 (62.6)	204 (74.5)	1	1
Assisted	09 (9.9)	32 (11.7)	1.01 (0.45-2.23)	1.54 (0.40-5.93)
CS	25 (27.5)	38 (13.9)	*2.35* (*1.31-4.22*)^@^	1.29 (0.44-3.76)
History of obstetric complication in current pregnancy				
Yes	54 (59.3)	66 (24.1)	*4.60* (*2.78-7.60*)^@^	*3.82* (*1.62-9.00*)^∗∗^
No	37 (40.7)	208 (75.9)	1	1
Maternal weight (kg)				
<50	17 (18.7)	27 (9.9)	*2.10* (*1.09-4.07*)^@^	2.68 (0.66-10.88)
≥50	74 (81.3)	247 (90.1)	1	1
Infant birth weight (gm)				
<2500	60 (65.9)	55 (20.1)	*7.71* (*4.56-13.02*)^@^	*5.58* (*2.39-13.03*)^∗∗∗^
≥2500	31 (34.1)	219 (79.9)	1	1
Presence of birth asphyxia				
Yes	41 (45.1)	52 (19)	*3.50* (*2.10-5.84*)^@^	1.21 (0.45-3.30)
No	50 (54.9)	222 (81)	1	1

^@^Candidate variables for multivariable analysis at *p* value ≤ 0.25. ^∗^Statistically significant at *p* ≤ 0.05 in multivariable logistic regression (for *p* value < 0.01^∗^ and <0.001^∗∗^). 1: reference; COR: crude odds ratio; AOR: adjusted odds ratio; CI: confidence interval.

## Data Availability

Our detailed data can be made available by contacting our e-mail address and the address of the funding organization for reasons of credibility.
